# Induction of a chemoattractant transcriptional response by a *Campylobacter jejuni *boiled cell extract in colonocytes

**DOI:** 10.1186/1471-2180-9-28

**Published:** 2009-02-04

**Authors:** Kenneth H Mellits, Ian F Connerton, Michael F Loughlin, Peter Clarke, Julie Smith, Eleanor Dillon, Phillippa L Connerton, Francis Mulholland, Christopher J Hawkey

**Affiliations:** 1 Division of Food Sciences, School of Biosciences, University of Nottingham, Sutton Bonington Campus, Loughborough, Leicestershire, LE12 5RD, UK; 2 MyCIB, School of Biosciences, University of Nottingham, Sutton Bonington Campus, Loughborough, Leicestershire, LE12 5RD, UK; 3 Wolfson Digestive Diseases Centre, University Hospital Nottingham, Nottingham, NG7 2UH, UK; 4 Institute of Food Research, Norwich Research Park, Colney, Norwich, NR4 7UA, UK

## Abstract

**Background:**

*Campylobacter jejuni*, the commonest cause of bacterial diarrhoea worldwide, can also induce colonic inflammation. To understand how a previously identified heat stable component contributes to pro-inflammatory responses we used microarray and real-time quantitative PCR to investigate the transcriptional response to a boiled cell extract of *Campylobacter jejuni *NCTC 11168.

**Results:**

RNA was extracted from the human colonocyte line HCA-7 (clone 29) after incubation for 6 hours with *Campylobacter jejuni *boiled cell extract and was used to probe the Affymetrix Human Genome U133A array. Genes differentially affected by *Campylobacter jejuni *boiled cell extract were identified using the Significance Score algorithm of the Bioconductor software suite and further analyzed using the Ingenuity Pathway Analysis program. The chemokines CCL20, CXCL3, CXCL2, Interleukin 8, CXCL1 and CXCL6 comprised 6 of the 10 most highly up-regulated genes, all with Significance Scores ≥ 10. Members of the Tumor Necrosis Factor α/Nuclear Factor-κB super-family were also significantly up-regulated and involved in the most significantly regulated signalling pathways (Death receptor, Interleukin 6, Interleukin 10, Toll like receptor, Peroxisome Proliferator Activated Receptor-γ and apoptosis). Ingenuity Pathway Analysis also identified the most affected functional gene networks such as cell movement, gene expression and cell death. In contrast, down-regulated genes were predominantly concerned with structural and metabolic functions.

**Conclusion:**

A boiled cell extract of *Campylobacter jejuni *has components that can directly switch the phenotype of colonic epithelial cells from one of resting metabolism to a pro-inflammatory one, particularly characterized by increased expression of genes for leukocyte chemoattractant molecules.

## Background

*Campylobacter jejuni *(*C. jejuni*) is a gram-negative micro-aerophilic bacterium responsible for the majority of human bacterial enteric infections worldwide [1,2]. *C. jejuni *is commonly found as a commensal organism in the intestinal tracts of a wide range of wild and domestic animals, including commercial poultry [3]. Cross-contamination from raw poultry or insufficient cooking of poultry meat are common sources of infection. Enteric infections by this pathogen are often associated with a potent localized inflammatory response. Symptoms arising from infection include watery or bloody diarrhoea with abdominal cramping and fever. In addition, *C. jejuni *can be invasive and is associated with septicaemia, meningitis, Guillain-Barré syndrome [4] and more recently with immuno-proliferative disease [5].

*C. jejuni *virulence factors for human disease include flagella based chemotaxis, adhesin-based cellular adherence, host cell invasion and the elaboration of a heat labile cytolethal distending toxin (CLDT) [2,6,7] In previous studies we have additionally shown that a heat stable *C. jejuni *boiled cell extract (BCE) is able to activate the transcription factor NF-κB (nuclear factor kappa-light-chain-enhancer of activated B cells) [8]. This signalling molecule is responsible for inducing the expression of a number of genes involved in inflammation and cell mediated immunity [9], including chemokines capable of attracting leukocytes, resulting in inflammation. NF-κB is held inactive in the cytoplasm of a cell, whilst its nuclear localization domain is masked by inhibitory IκB proteins. If IκB is phosphorylated, leading to ubiquitin-mediated proteolysis, then NF-κB is released to transport to the nucleus of the cell, where it affects transcription of κB-responsive promoters. Therefore products that activate NF-κB can be presumed to have a strong role in triggering inflammation. Previous work has shown that live *C. jejuni *and a BCE can induce both NF-κB, and the synthesis and release of the chemokine interleukin-8 [8].

In order to identify a wider range of genes affected by C. *jejuni *products and assess the relative importance of the NF-κB response we used microarray technologies to identify genes that were both up and down-regulated in HCA-7 cells after exposure to a C. *jejuni *BCE [8,10]. Use of the Ingenuity Pathway Analysis (IPA) program suite enabled us to group co-regulated genes in order to identify the cellular signalling pathways activated in HCA-7 cells in response to C. *jejuni *BCE. The transcriptomic data were confirmed by real time quantitative PCR (RQ-PCR).

## Methods

### *C. jejuni *culture and preparation of BCE

The type strain *C. jejuni *National Collection of Type Cultures (NCTC) 11168 was used throughout these experiments, since it was originally isolated from a patient with diarrhoea, its genome sequence is available and it has a well-characterized pathological phenotype [11]. It was incubated on blood-agar plates (Blood Agar Base CM0271 from Oxoid, Basingstoke, UK with 5%, v/v defibrinated horse blood) under micro-aerobic conditions for 24 h. and used to inoculate Nutrient Broth no. 2 (Oxoid CM0067, 600 ml in 1000 ml flask). Inoculated flasks were shaken at 140 rpm at 42°C for 16 h. under micro-aerobic conditions. Culture purity was determined by plating samples from each overnight culture onto blood plates and incubating for 24 h., 42°C in micro-aerobic conditions. Bacteria were collected by centrifugation at 10,000 *g *for 15 min. The cell pellet was washed three times in Phosphate Buffered Saline (PBS), weighed and re-suspended in PBS to achieve a 10% (w/v) suspension, which was boiled for 10 min., cooled on ice for 5 min. before being centrifuged at 10, 000 *g *for 10 min. The supernatant was collected, passed through a 0.2 μm filter to remove residual bacteria and stored at -20°C until required.

### HCA-7 cell culture and treatment with *C. jejuni *BCE

The human colonocyte line HCA-7 [10], clone 29, was grown to confluence in a 5% CO_2 _atmosphere in monolayer cultures on monolayer dishes in Dulbecco's Modified Eagle's Medium supplemented (DMEM) with 100 μg/ml penicillin, 100 μg/ml streptomycin and fetal calf serum at 10% (v/v, Fisher Scientific, Loughborough, UK) at 37°C. Twenty-four hours prior to induction by BCE, HCA-7 cells were transferred to serum-free DMEM. HCA-7 cells were then incubated for 6 h. with 25 μl BCE or PBS control in a total volume of 1 ml of DMEM. The BCE preparation was determined in parallel to induce NF-κB 300-fold using a reporter cell assay [8]. At 6 h. post induction total RNAs were extracted using RNAeasy columns (Qiagen, West Sussex, UK). Total RNA yields and purity were determined using an Agilent 2100 Bioanalyzer (Agilent Technologies UK Limited, Stockport, UK).

### cDNA synthesis

Approximately 10 μg of total RNA was reverse transcribed at 42°C for 1 h. to generate first strand DNA using 100 pmol oligo dT_(24) _primer containing a 5'-T7 RNA polymerase promoter sequence (5'-GCCAGTGAATTGTAATACGACTCACTATAGGGAGGCGG-(dT)_24_-3'), 50 mM Tris-HCl (pH 8.3), 75 mM KCl, 3 mM MgCl_2_, 10 mM dithiothreitol (DTT), 10 mM dNTPs and 200 units SuperScript II reverse transcriptase (Invitrogen Life Technologies, Strathclyde, UK). Second strand DNA synthesis was carried out at 16°C for 2 h., using 10 units of *E. coli *polymerase I, 10 units of *E. coli *DNA ligase and 2 units of RNase H in a reaction containing 25 mM Tris-HCl (pH 7.5), 100 mM KCl, 5 mM MgCl_2_, 10 mM (NH_4_)SO_4_, 0.15 mM β-NAD^+ ^and 10 mM dNTPs. 10 units of T4 DNA polymerase were added and the reaction allowed to proceed for a further 5 min. before termination with 0.5 M EDTA. Double stranded cDNA products were purified using the GeneChip Sample Cleanup Module (Affymetrix, Santa Clara, CA, USA).

### cRNA synthesis

The synthetic cDNAs were *in vitro *transcribed using T7 RNA polymerase (ENZO BioArray High Yield RNA Transcript Labeling Kit, Affymetrix, Santa Clara, CA, USA) with biotinylated ribonucleotides to generated biotinylated complementary RNAs (cRNAs). The cRNAs were purified using the GeneChip Sample Cleanup Module before random fragmentation at 94°C for 35 min. in a buffer containing 40 mM Tris-acetate (pH 8.1), 100 mM potassium acetate and 30 mM magnesium acetate to generate molecules of approximately 35 to 200 bases long.

### Array hybridization

Changes in gene transcription were analyzed by hybridization to Affymetrix Human Genome U133A array (HG-U133A) which contains probes for over 22,000 transcripts, including representation of the RefSeq database sequences and probe sets http://www.affymetrix.com/products_services/arrays/specific/hgu133.affx. The fragmented cRNAs were mixed with 0.1 mg/ml of sonicated herring sperm DNA in a hybridization buffer containing 100 mM 2-N-morpholino-ethane-sulfonic acid (MES), 1 M NaCl, 20 mM EDTA and 10% Tween 20 to make the hybridization mixture. The hybridization mixture containing the fragmented cRNA was denatured at 99°C for 5 min. and equilibrated for a further 5 min. at 45°C before centrifugation at 10,000 *g *for 5 min. to remove any insoluble material from the hybridization mixture. The hybridization mix was transferred to the ATH1-121501 genome array (Affymetrix, Santa Clara, CA, USA) cartridge and hybridized at 45°C for 16 h. on a rotisserie at 60 rpm.

After a 16 h. hybridization period the arrays were washed and stained in a Fluidics station (Affymetrix, Santa Clara, USA). The arrays were initially washed in a low stringency buffer A (6 × SSPE [0.9 M NaCl, 0.06 M NaH_2_PO_4_, 0.006 M EDTA], 10% Tween 20) at 25°C for 10 min. and then incubated with a high stringency buffer B (100 mM MES, 0.1 M NaCl, 10% Tween 20) at 50°C for 20 min. and stained with 10 mg/ml of streptavidin phycoerythrin (SAPE), in stain buffer containing 100 mM MES, 1 M NaCl, 0.05% Tween 20 and 2 mg/ml BSA at 25°C for 10 min. After a further wash in wash buffer A at 25°C for 20 min. they were stained with biotinylated anti-streptavidin antibody at 25°C for 10 min. After antibody staining the arrays were stained again with SAPE for signal amplification and washed with buffer A at 30°C for 30 min. The arrays were finally scanned and the intensities averaged with the Agilent GeneArray Scanner (Agilent Technology UK, West Lothian, UK).

### Statistical analysis of Array data and Generation of Networks and Canonical Pathways

In order to identify genes of interest we used the S Score (Significance Score) algorithm as implemented in the Bioconductor software suite http://www.bioconductor.org[12] based on the R package http://www.r-project.org[13] that takes advantage of the fact that most genes are unchanged and calculates an S score (SD from the mean). The S score threshold of +/- 2.5 and an alpha value of P = 0.005 was used to define gene changes of interest. Data listing all genes that satisfied these criteria were analyzed by Ingenuity Pathway Analysis, Ingenuity^® ^Systems, http://www.ingenuity.com. This generated functional networks and canonical pathways that connect the differentially expressed genes, using the IPA Knowledge base, where the interactions are supported by peer reviewed publications and which contains over 1.4 million interactions between genes, proteins, and drugs. Scores were assigned allowing ranking of the networks, using a Fisher's right tailed exact test.

### Analysis of microarray data by real time quantitative PCR

To confirm microarray results, extracted HCA-7 total RNA was amplified by oligo dT(15) primers according to the Im-Prom II Kit (Promega UK, Southampton UK) methodology. Representative samples of genes from a number of the major functional groups and gene networks identified by IPA program were selected to confirm the array data using RQ-PCR analysis (Tables 1, 2 and 4) under appropriate conditions for an ABI Prism 7700. Primer and probe design utilized Primer Express software (Applied Biosystems, Warrington, UK). The primers were validated for gene specificity by agarose gel electrophoresis. Reporter dye-labelled probes were used with FAM (6-carboxyfluorescein) at the 5'-end and TAMRA (6-carboxy-tetramethyl-rhodamine) at the 3'-end. Reactions were set up in a final volume of 25 μl containing 12.5 μl of 2 × Taqman Universal PCR Mastermix (Applied Biosystems, Warrington, UK): 0.75 μl of each primer (10 pmol/μl), 0.5 μl of probe (10 pmol/μl), 2 μl of cDNA (equivalent to 5 ng total RNA/μl) and 8.5 μl of water. Samples were analyzed in triplicate and the emission released reporter dye was monitored by an ABI Prism 7700 Sequence Detector (Applied Biosystems, Warrington, UK) using the default PCR program of 2 min at 50°C and 10 min at 95°C; each cycle included denaturing at 95°C for 15 s and annealing at 60°C for 1 min. Analysis of the data was via the Sequence Detection System (SDS) software (Applied Biosystems, Warrington, UK). A no template control was included in each analysis and did not give any signal with any of the primer/probe combinations. RQ-PCR data were normalized using primers to β-actin based on the considerations outlined by Hugget *et al*. [14].

**Table 1:** Primers and probes used in the study

Gene	Forward Primer	Reverse Primer	Probe
β-actin	TCACCGAGCGCGGCT	TAATGTCACGCACGATTTCCC	CAGCTTCACCACCACGGCCGA

Interleukin-8	ATTTTCCTAGATATTGCACGGGAG	GCAAACCCATTCAATTCCTGA	AAAATTGAGGCCAAGGGCCAAGAGAA

ATPase, Na+/K+ transporting, Beta1 polypeptide	GCCCAGAGGGATGACATGAT	CAGACCTTTCGCTCTCCTCG	TTTGAAGATTGTGGCGATGTGCCCA

Syndecan 4	TGGGTGGTTGAGTGAGTGAATT	CCTCAACTATTCCAGCCCCAT	TTTCTCTTGCCCTGTTCCTGGTGCC

Retinoic acid receptor responder (tazarotene induced) 1	ACCCTGAGGAACCTGCTGGT	TGGTTTTTTGTTTCTCAGTCTGCT	TGAGCAGAGTTCAGTGTGCATGCGCT

tumor necrosis factor, alpha-induced protein 3	CTTTGAGTCAGGCTGTGGGC	TTGGATGCAATTCCTTCTTTCC	ACCACAGGGAGTAAATTGGCCTCTTTGATACA

nuclear factor of kappa light polypeptide gene enhancer in B-cells inhibitor, alpha	GGCCTCCAAACACACAGTCA	GCTGCCAGAGAGTGAGGATGA	CTCCGTGAACTCTGACTCTGTGTCATAGCTCTC

matrix metallo-peptidase 7	GATCCCCCTGCATTTCAGG	CTGGCCCATCAAATGGGTAG	TCATGATTGGCTTTGCGCGAGG

Forward primer, reverse primer and Taqman probes for RQ-PCR assays used, all listed 5' - 3' direction.

**Table 2:** Up-regulated genes. Functional classes of genes shown are ordered by the S score of the most highly regulated examples in the class with S score ≥ 5.

Function	Symbol	Name	S Score
Chemokine	CCL20	Chemokine (C-C Motif) Ligand 20	13.542
	
	CXCL3	Chemokine (C-X-C Motif) Ligand 3	11.866
	
	CXCL2	Chemokine (C-X-C Motif) Ligand 2	11.742
	
	IL8	Interleukin 8	11.393
	
	CXCL1	Chemokine (C-X-C Motif) Ligand 1	11.096
	
	CXCL6	Chemokine (C-X-C Motif) Ligand 6	10.79
	
	CCL2	Chemokine (C-C Motif) Ligand 2	5.294

TNF/NFkB superfamily	TNFAIP3	Tumor Necrosis Factor, Alpha-Induced Protein 3	11.678
	
	IKBA	Nuclear factor of kappa light polypeptide gene enhancer in B-cells inhibitor, alpha	10.956
	
	TNIP1	TNFAIP3 Interacting Protein 1	9.344
	
	TNFAIP2	Tumor Necrosis Factor, Alpha-Induced Protein 2	8.293
	
	OPTN	Optineurin	6.487
	
	IL32	Interleukin 32	6.12
	
	NFKB1	Nuclear Factor Kappa B (P105)	5.355

Apoptosis/Cell death	UBD	Ubiquitin D	11.647
	
	BIRC3	Baculoviral IAP Repeat-Containing 3	11.063
	
	CFLAR	CASP8 And FADD-Like Apoptosis Regulator	6.224
	
	SGK	Serum/Glucocorticoid Regulated Kinase	5.705
	
	ISG20	Interferon Stimulated Exonuclease Gene 20 kda	5.575

Extracellular Matrix	MMP7	Matrix Metallopeptidase 7 (Matrilysin, Uterine)	9.812
	
	SDC4	Syndecan 4 (Amphiglycan, Ryudocan)	8.923
	
	LAMA3	Laminin, Alpha 3	5.824
	
	LAMC2	Laminin, Gamma 2	5.32

Folate receptor	FOLR1	Folate Receptor 1 (Adult)	8.963

Redox state	SOD2	Superoxide Dismutase 2, Mitochondrial	8.879
	
	TXNRD1	Thioredoxin Reductase 1	6.378

Cell adhesion	ICAM1	Intercellular Adhesion Molecule 1	8.879
	
	FNDC3B	Fibronectin Type III Domain Containing 3B	5.851

Cytokines/Receptors	IFNGR1	Interferon Gamma Receptor 1	8.403
	
	CSF2	Colony Stimulating Factor 2	5.101
	
	PLAT	Plasminogen Activator, Tissue	7.464
	
	SERPINB2	Serpin Peptidase Inhibitor 2	6.319

Energy metabolism	ATP1B1	Atpase, Na+/K+ Transporting, Beta 1 Peptide	7.184
	
Nuclear transcription	CEBPD	CCAAT/Enhancer Binding Protein Delta	6.708
	
	RARRES1	Retinoic Acid Receptor Responder	6.179

Antibacterial	LCN2	Lipocalin 2	6.6
	
	PI3	Peptidase Inhibitor 3 (Elafin)	5.057

Cell signalling	CDC42	Cell Division Cycle 42	7.28
	
	DUSP5	Dual Specificity Phosphatase 5	6.541
	
	SGPL1	Sphingosine-1-Phosphate Lyase 1	6.242

Cytoskeleton/cytokinesis	TPM1	Tropomyosin 1	5.689
	
	PDLIM5	PDZ And LIM Domain 5	5.169

Transcription, protein synthesis and export	SF3B1	Splicing Factor 3b, Subunit 1,	5.146
	
	UGCG	UDP-Glucose Ceramide Glucosyltransferase	5.388
	
Cell cycle	PLK2	Polo-Like Kinase 2	5.55

Structural	SYNGR3	Synaptogyrin 3	5.133

Antigen presentation	TAP1	Transporter 1, ATP-Binding Cassette	5.207

### Chemokine and cytokine analyses

Cultured cells were prepared and induced as described above. After 6 h. incubation, the media was removed and stored at -20°C until examined using a Coulter-Alter Flow Cytometer in conjunction with a BD cytometric bead array human inflammation kit according to manufacturer's instructions (BD Biosciences, Oxford, UK). IL8 and CCL20 (MIP-3α) were specifically measured using a sandwich ELISA, by capture with a murine anti-human IL8 or CCL20 and detected using biotinylated goat anti-human IL8 using streptavidin-coupled horseradish-peroxidase, according to the manufacturer's instructions (R&D Systems, Minneapolis, MN, USA).

## Results

The Bioconductor and IPA programs identified 356 genes that changed with a positive or negative S score of 2.5 or greater (maximum 13.54). Three hundred were up-regulated and 56 were down-regulated (Additional file 1).

### Up-regulated genes

Table 2 shows 48 genes that were up-regulated with an S score of 5 or greater. These were grouped by class and ordered by the highest S score in each class. Chemokines dominate the most highly up-regulated genes with six of the ten highest S scores. Members of the TNFα-NF-κB super family were also highly up-regulated (Table 2). Other highly up-regulated genes were those involved in apoptosis and ubiquitination, extra-cellular matrix proteins, the folate receptor, superoxide dismutase, thioredoxin reductase, Intercellular Adhesion Molecule (ICAM) 1 and cytokines or their receptors (Colony Stimulating Factor [CSF] 2 and interferon-γ receptor 1).

### Down-regulated genes

Fewer genes were down-regulated than those that were up-regulated and negative S scores were less pronounced than those for the up-regulated genes. For comparative purposes Table 3 shows down-regulated genes that were selected on the basis of a more permissive S score of -2.6 or less to yield a similar number (46). These genes were grouped by class and ordered by the highest negatively regulated (lowest value) S score in each class. The pattern of down-regulated gene classes differ markedly to those that were up-regulated. Most prominent were genes concerned with the maintenance of normal cell cycle, DNA replication and cell structure. The down-regulated group feature specific genes encoding components involved in membrane transport, mitosis, nucleotide synthesis, transcription, protein synthesis and export, membrane transport and energy metabolism.

**Table 3:** Down-regulated genes Functional classes of genes shown are ordered by the S score of the most highly regulated examples in the class with S score ≤ -2.6.

Function	Symbol	Name	S Score
Cell cycle, DNA replication and Mitosis	ID1	Inhibitor Of DNA Binding 1	-4.416
	
	ID3	Inhibitor Of DNA Binding 3	-4.304
	
	ID2	Inhibitor Of DNA Binding 2	-4.054
	
	LHX3	LIM Homeobox 3	-3.181
	
	KLF1	Kruppel-Like Factor 1	-2.97
	
	FOXF2	Forkhead Box F2	-2.684
	
	SFN	Stratifin	-4.086
	
	FGFBP1	Fibroblast Growth Factor Binding Protein 1	-3.922
	
	SKP2	S-Phase Kinase-Associated Protein 2 (P45)	-3.035
	
	RPA3	Replication Protein A3	-2.975
	
	RFC4	Replication Factor C 4	-2.845
	
	SPBC25	Spindle Pole Body Component 25 Homolog	-2.688

Structural	REG1A	Regenerating Islet-Derived 1 Alpha	-4.213
	
	CX36	Connexin-36	-3.79
	
	COL4A5	Collagen, Type IV, Alpha 5	-3.69
	
	ODF1	Outer Dense Fiber Of Sperm Tails 1	-3.511
	
	CD248	CD248 Molecule, Endosialin	-2.965

Membrane transport	SLC2A1	Solute Carrier Family 2, Member 1	-3.912
	
	CRIP1	Cysteine-Rich Protein 1 (Intestinal)	-3.079
	
	SCNN1A	Sodium Channel, Nonvoltage-Gated 1 Alpha	-2.918
	
	HFE	Hemochromatosis Gene	-2.723

Transcription, protein synthesis and export	CHMP6	Chromatin Modifying Protein 6	-3.599
	
	RANBP1	RAN Binding Protein 1	-3.48
	
	EHBP1	EH Domain Binding Protein 1	-3.106
	
	RRM2	Ribonucleotide Reductase M2 Polypeptide	-2.957
	
	CTDSPL	Small Carboxy-Terminal Domain Phosphatase	-2.838
	
	DARS2	Aspartyl-Trna Synthetase 2 (Mitochondrial)	-2.795
	
	POLR3K	Polymerase (RNA) Subunit K	-2.701

Nucleotide synthesis	UNG	Uracil-DNA Glycosylase	-3.553
	
	GLRX	Glutaredoxin	-3.325
	
	DUT	dUTP Pyrophosphatase	-2.967
	
	TYMS	Thymidylate Synthetase	-2.687

Energy metabolism	ATAD4	ATPase Family, AAA Domain Containing 4	-3.185
	
	COX7B	Cytochrome C Oxidase Subunit 7B	-2.893

Cytoskeleton/cytokinesis	M-RIP	Myosin Phosphatase-Rho Interacting Protein	-2.954
	
	MALL	Mal, T-Cell Differentiation Protein-Like	-2.918
	
	ARHGAP29	Rho Gtpase Activating Protein 29	-2.909
	
	ROCK2	Rho-Associated, Coiled-Coil Containing Protein Kinase 2	-2.701

Cytokine	TGFB2	Transforming Growth Factor, Beta 2	-2.909
	
	C1QTNF3	C1q And TNF Related Protein 3	2.701

Protease	SPINK1	Serine Peptidase Inhibitor, Kazal Type 1	-2.889

Cell adhesion	LGALS4	Galectin 4	-2.869

Redox	TXNIP	Thioredoxin Interacting Protein	-2.843

Cell signalling	HS1BP3	HS1-Binding Protein 3	-2.755

Anti-inflammatory	ANXA1	Annexin A1 (Lipocortin 1)	-2.703

Matrix	LAMB1	Laminin, Beta 1	-2.702

### Signalling pathways

**Figure 1:**
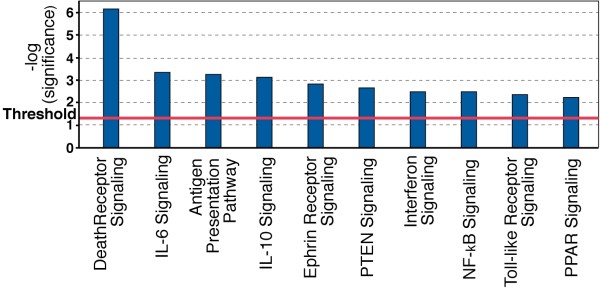
**Canonical Signalling Pathways identified by IPA software as significantly regulated by *C. jejuni *BCE**. A Fisher's exact test was used to calculate a *p*-value (Bars) determining the probability that the association between the genes in the dataset and the canonical pathway can be explained by chance alone. Threshold refers to the cut off for p < 0.05.

**Figure 2:**
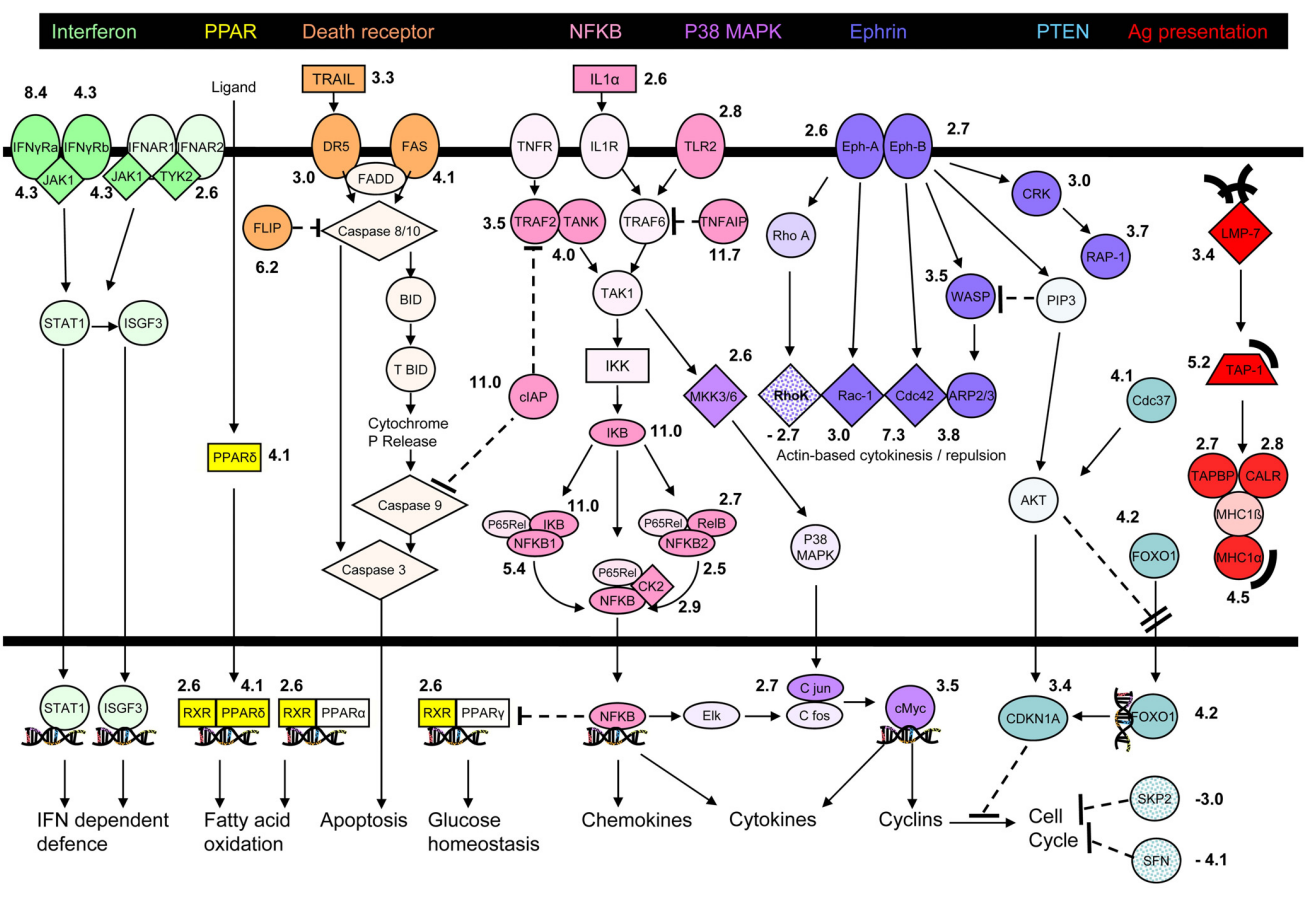
**Regulated molecules in canonical signalling pathways identified by IPA**. Individual pathways are identified by colours assigned in the black-backed heading at the top. Significantly up-regulated genes are shown in darker colour. Significantly down-regulated genes are shown stippled. Numerical values beside regulated genes show the S score. All genes identified by the IPA programme as significantly regulated have been included, together with a limited number of non-regulated genes to portray a simplified view of pathway continuity. For simplicity, molecules participating in more than one pathway are arbitrarily shown once in the most dominant pathway. Common transcriptional and other consequences of pathway activation are indicated in the Figure. Symbols are as in Figure See Figure 3 except that **---l **= Inhibition (direct or indirect), **---ll **= blocks translocation,**)** = Peptide, double helix = transcription.

**Figure 3:**
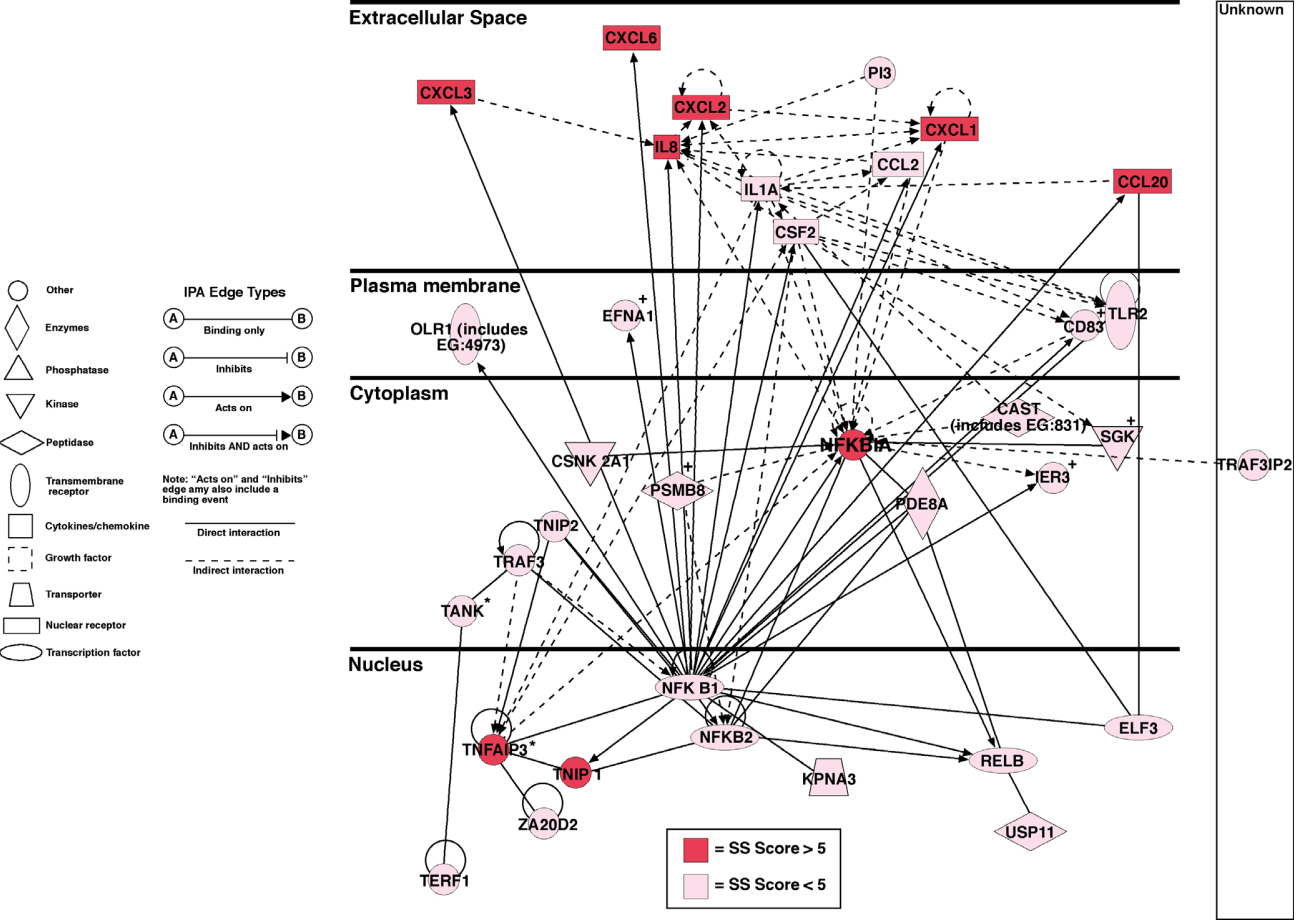
**IPA generated NF-κB-centred gene network**. Network contains nodes (gene/gene product) and edges (indicating a relationship between the nodes) showing the cellular/subcellular location as indicated. An asterisk indicates that duplicates were identified in each dataset. Function classes of nodes indicated by shape to represent functional class, a plus sign indicates node is contained in other networks. All 35 focused genes are significantly up-regulated. Genes with an S score of ≥ 7 are shown in red and those with an S score of between 2.5–7 are shown pink. Explanation of edge types and shapes is indicated.

IPA identified a number of canonical signalling pathways that were most significantly affected (Figure 1). Figure 2 shows a simplified composite of all genes identified by IPA as being part of specific signalling pathways that are most significantly regulated, together with their individual S scores. Here the central mediator is the NF-κB signalling pathway that is clearly contributory in affecting the signalling through the Death Receptor, IL6, IL10, Toll-like receptor and PPAR pathways (also see Gene Networks section below and Figure 3 which also features NF-κB). In addition, several other canonical signalling pathways, some of which do not feature NF-κB, were also identified as significantly affected.

The antigen presentation pathway was identified through up-regulation of the Large Multifunctional Protease (LMP)-7, Transporter Associated with Antigen Processing (TAP) 1, TAP-binding protein (TAPBP), Calreticulin (CALR) and the Major Histocompatibility Complex (MHC)1-α.

Activation of the interferon-γ receptor defence signalling pathway was noted through up-regulation of both components of interferon-γ receptor, Janus kinase (JAK) 1 and Tyrosine Kinase (TYK) 2.

Activation of the ephrin signalling pathway, indicating activation of actin-based cytokinesis and repulsion. The pathway included up-regulation of ephrin receptor sub components, RHO family, GTP binding protein (Rac1), Cell Division Cycle (CDC) 42, Wiskott-Aldrich syndrome protein (WASP), actin-related protein 2 (ARP2), V-crk homologue (CRK) and Ras oncogene family member (RAP)1B with rho-associated coiled-coil containing protein kinase (ROCK) 2.

Finally, up-regulation of most components of the PI3K-phosphatase signalling pathway were noted, including phosphatase and tensin homology (PTEN) pathway indicating possible effects on the cell cycle, including Cell Division Cycle (CDC) 37, Forkhead Box (FOX)O1A and Cyclin Dependent Kinase Inhibitor (CDKN)1a (P21). SFN (Stratifin or 14-3-3σ) however, was down-regulated.

### Predicted functional effects

**Figure 4:**
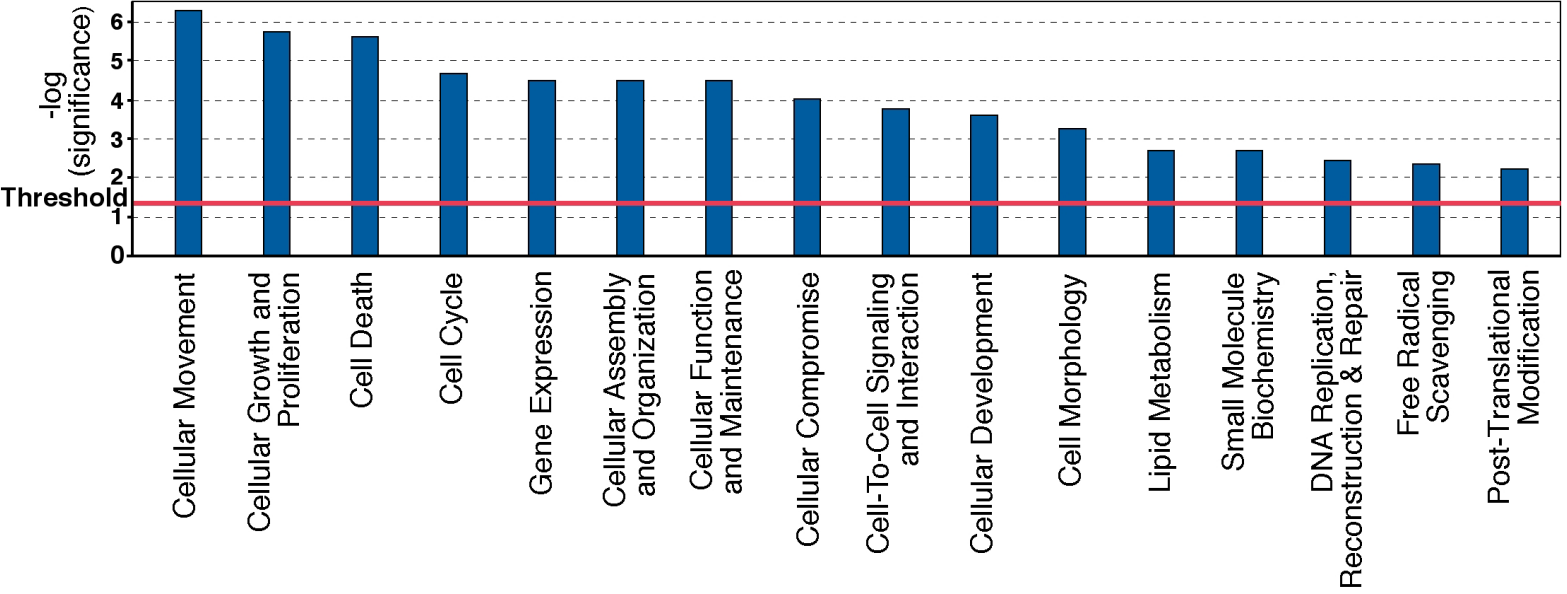
**Functional Molecular and Cellular pathways significantly affected by *C. jejuni *BCE**. A Fisher's exact test was used to calculate a *p*-value (Bars) determining the probability that the association between the genes in the dataset and the canonical pathway can be explained by chance alone. Threshold refers to the cut off for p < 0.05.

The IPA program can determine if groups of significantly changed genes have related cellular and molecular functions (Figure 4). Here IPA identified 16 functional categories that were significantly affected by the *C. jejuni *BCE. The most prominent functions implicated were cellular movement (reflecting changes in chemokines, adhesion receptors and molecules affecting cytokinesis), cell growth and proliferation and cell death.

### Gene networks

The IPA program constructed 16 interconnected gene networks that were significantly altered as a result of treatment of HCA-7 cells with *C. jejuni *BCE, all with network scores of ≥ 8. The network score is the probability that a network would be assembled by chance where a level of > 3 is statistically significant, at p < 0.001. In the four most significantly regulated all 35 focus genes of the network were affected, all giving an identical score of 52 (*P *< 1E-52).

The first network (Figure 3) contains genes concerned with cellular movement, particularly chemotaxis. NF-κB occupies a central position in the network and includes a number of genes which are known to up-regulate including a number of chemokines.

The second network (Additional file 2) likewise contains genes associated with cellular movement, including cytokinesis and inflammatory responses. Up-regulated genes include Ephrin Receptor B2 (EPHB2), PTGS2 (COX-2), ICAM1, both components of interferon-γ receptor, IL23A, IL27RA, JAK1, JUNB proto oncogene, Mitogen Activated Protein Kinase Kinase Kinase Kinase (MAP4K4), TYK2, Mothers Against DPP homologues (SMAD) 3, with 2 genes shown to be significantly down-regulated (SH2B and Transforming Growth Factor [TGF] β2).

MYC occupies a central position in the third network (Additional file 3), which contains genes concerned with the regulation of the cell cycle. Up-regulated genes include MYC as well as FAS, folate receptor (FOLR1), HLA molecules E, F and G, laminins β3, α3 (LAM-B3, A3) and γ2 (LAMC2), Matrix Metallo Proteinase (MMP)7, and SOD2. Down-regulated were Laminin β1 (LAMB1), RAN Binding Protein 1 (RANBP1) Thioredoxin Interacting Protein (TXNIP) and Thymidylate Synthetase (TYMS).

Finally, a network (Additional file 4) contains genes affecting cell death and gene expression. The network contains 25 genes that were up-regulated, including Activating Transcription Factor (ATF) 3, cellular Inhibitor of Apoptosis Proteins (cIAP) 1 and 2 (BIRC 2 and 3), cyclin dependent kinase (CDK) 7, cyclin dependant kinase inhibitor (CDKN) 1A, GATA binding protein (GATA) 6, TNFα-Induced Protein (TNFAIP) 2, the TNF-Related Apoptosis-Inducing Ligand (TRAIL or TNFSF10), its receptor TRAILR2 (TNFRSF10B or Death receptor [DR] 5) and TNF Receptor Associated Factor (TRAF) 2. Whilst CDKN1A is up-regulated, CDKN3 is down-regulated, as are the Inhibitors of DNA Binding (ID)1,2 and 3, Mini-Chromosome Maintenance homologue (MCM) 6, RCF4, rho-associated, coiled-coil containing protein kinase (ROCK) 2 and S-Phase Kinase-Associated Protein (SKP) 2.

### Validation of Microarray data

Changes in gene expression identified by microarray were confirmed by RQ-PCR (Table 4). However, hierarchical differences are apparent between the RQ-PCR values normalized against β-actin compared with the S score associated with the significantly regulated genes as indicated by differential hybridization of the cRNA preparations to the microarray.

**Table 4:** Comparison of results for selected up-regulated genes determined by Affymetrix/S score and RQ-PCR.

Gene Description	Ingenuty Name	Affymetrix Probe Set	S Score	Fold RQ-PCR	Network	Location
Interleukin-8	IL8	211506_s_at	11.393	59.4 ± 15.5	See Figure 3	Extra-cellular

ATPase, Na+/K+ transporting, Beta 1 polypeptide	ATP1B1	201242_s_at	7.184	4.5 ± 1.8	10	Plasma Membrane

Syndecan 4	SDC4	202071_at	8.823	4.0 ± 0.84	5	Plasma Membrane

Retinoic acid receptor responder (tazarotene induced) 1	RARRES1	221872_at	6.179	2.4± 0.7	8	Plasma Membrane

tumor necrosis factor, alpha-induced protein 3	TNIP1	207196_s_at	9.344	2.0 ± 0.2	See Figure 3	Nucleus

nuclear factor of kappa light polypeptide gene enhancer in B-cells inhibitor, alpha	NFKBIA	201502_s_at	10.956	4.0 ± 1.2.	See Figure 3	Cytoplasm

Matrix Metallo-peptidase 7	MMP7	202644_s_at	9.812	2.1 ± 4.2	9 & See Additional file 3	Extra-cellular

For each gene ingenuity description, name and Affymetrix probe set, assigned network and cellular location are shown together with the S score and fold RQ-PCR change compared to β-actin control.

### Chemokine and cytokine responses

To further validate the gene transcriptional changes using microarray and RQ-PCR methods, we measured the levels of secretory immunomodulatory proteins in parallel cell supernatants of HCA-7 cells pre- and post-induction with *C. jejuni *BCE. Table 5 presents the chemokine and cytokine levels of pro- and anti-inflammatory secretory proteins. Consistent with the microarray observations the pro-inflammatory chemokine CCL20 showed a 12.6-fold increase in levels 6 h. post treatment. IL8 levels were also found to increase, but far more dramatically than CCL20 with a 460-fold induction. HCA-7 colonocytes are particularly IL8 responsive with post-induction levels of 18.4 ng/ml, an observation that is consistent with previous reports with this cell line [8]. The pro-inflammatory cytokine IL1β showed a weak response consistent with the transcriptional response recorded in the microarray study. Pro-inflammatory cytokine IL6 showed a 5-fold increase, whereas the anti-inflammatory cytokine IL10 remained static. The transcriptional response of the genes encoding IL6 and IL10 did not show marked transcriptional changes but the pathways associated with these immunomodulatory proteins were recognized by IPA and are responsive to NF-κB.

**Table 5:** Cytokine and chemokine levels (pg/ml) pre- and post-induction of HCA-7 cells with *C. jejuni *BCE for 6 h.

	Pre-Induction	Post-Induction	Fold-Induction
IL10	12 (± 2)	15 (± 3)	1.25
IL6	30 (± 3)	150 (± 5)	5
IL1β	20 (± 4)	30 (± 6)	1.5
IL8	40 (± 16)	18,400 (± 400)	460
CCL20	30 (± 6)	380 (± 40)	12.6

## Discussion

Understanding the pathogenesis of *C. jejuni *enteric disease is important both because *C. jejuni *is a major cause of diarrhoeal illness worldwide and because it may serve as a model for ulcerative colitis, the pathology of which it closely resembles [15]. Previous work has shown that direct interaction between *C. jejuni *and epithelial cells is capable of inducing pro-inflammatory and pro-secretory processes [8,16]. These are associated with cellular invasion [17] and secretion of IL8 by CLDT dependent and independent mechanisms [16,18]. Direct use of a BCE has allowed us to use a reductionist approach to investigate effects of *C. jejuni *that are not dominated by these linked processes of cellular invasion by live bacteria and by toxin based cell lysis. BCE has been determined to contain polysaccharide and protein components of the cell. As demonstrated previously the NF-κB inducing activity of *C. jejuni *BCE is relatively insensitive to digestion by protease K [8]. However the protein content has been determined using tryptic digests of SDS-polyacryamide extracted protein bands using MALDI-TOF mass spectrometry as flagellin (Cj1339c), trigger factor (Cj0193c), lipoprotein (Cj0983), major outer membrane protein (Cj0599), cytochrome-c peroxidase (Cj0358), bacterioferritin (Cj1534c), cell binding factor PEB4A (Cj0496), hypothetical protein (Cj0706), periplasmic protein (Cj0772c), fibronectin binding protein (Cj1478c), non-heme iron protein (Cj0012c), periplasmic protein (Cj1380), periplasmic protein (Cj0420), periplasmic protein (Cj0998c), DNA-binding protein HU (Cj0913c), periplasmic cytochrome C (Cj1153) and thioredoxin (Cj0147c) [11]. The polysaccharide component features α-glucan oligomers. The *C. jejuni *extract is notably devoid of the dominating heat-labile effects of the CLDT. *C. jejuni *BCE, like infection with live *C. jejuni*, has been shown to be a potent inducer of NF-κB using either luciferase based reporter assays, western blots with antibodies against IκB or electrophoretic mobility shift assays in epithelial cells [8] but, unlike treatment with live *C. jejuni*, this does not lead to host cell lysis. These observations are consistent with the hypothesis that a heat stable component plays a significant role in the pro-inflammatory response upon exposure to *C. jejuni*.

We hypothesize that NF-κB modulation is central to the response of enterocytes to *C. jejuni *BCE; to study this we determined the global changes in gene expression induced by *C. jejuni *BCE treatment of the well-differentiated human colonocyte line HCA-7, clone 29. In order to ensure the relevance of our results we have adopted stringent criteria for the identification of significantly affected genes and used the IPA program to determine the functional links between these gene products, identify the signalling pathways and networks to which they belong. These changes were validated by showing similar affects on mRNA levels when genes of interest were investigated by real-time quantitative PCR.

Consistent with the initial hypothesis that NF-κB plays a major role in the response of HCA-7 cells to *C. jejuni *BCE, and features in 8 of the 11 designated signalling pathways identified by IPA as up-regulated. Moreover, all genes in the NF-κB associated network (Figure 3) were up-regulated by *C. jejuni *BCE. The dominant component of this response concerned up regulation of chemokines that would act to induce the influx of acute inflammatory cells that characterize *Campylobacter *colitis. Our data are remarkably similar to transcriptomic data reported by Hinata *et al*., who activated NF-κB by transfecting clones expressing subunits of NF-κB to show up-regulation of the chemokines CXCL3 (GRO3) IL8, CXCL6, CXCL2 (GRO2), CXCL20 (SCYA20), CXCL1 (GRO1), CCL2 (CXYA2) as well as IL1α and CSF2, all of which were also significantly up-regulated in our study [19]. The NFKB1, NFKB2 and RELB components of NF-κB are also similarly up-regulated in our study. Other changes that are likely to be of functional importance and are the up-regulation of COX2 (PTGS2), TNIP2, MYC, SOD2, ELF3 and ICAM1 (Additional file 1), where all of these processes are also downstream targets of NF-κB [20] and mediators of feedback inhibition of NF-κB activation such as NFKBIA (IκB) [9], TNIP1 [21] and TNIP2 (Figure 3) [22]. A central role for NF-κB is also supported by data using the monocytic cell line THP-1 [23]. Studies in which Caco-2 cells were incubated with live bacteria resulted in expression of many genes similar to those reported here, including chemokines, but additionally, the NF-κB inhibitor NFKBIZ [24]. This difference may reflect the ability of live bacteria to invade cells and/or elaborate a CLDT with DNase activity [6].

The pattern of significantly down-regulated genes (Table 3) is remarkably different with a reduction in expression in constitutively expressed genes concerned with nucleotide synthesis, transcription, DNA replication, mitosis, structural protein synthesis, membrane transport and energy metabolism. These changes likely reflect the reprioritization of cellular metabolism in response to pro-inflammatory products.

Whether the changes caused by the *C. jejuni *BCE would lead to increased or reduced apoptosis is difficult to predict, especially as HCA-7 lack a functional TP53 protein, although these cells are capable of apoptosis given the appropriate signal [25]. Invasive *C. jejuni *infection can cause cell death in HCA-7 cells [16], although we did not see this with the addition of BCE [8]. Increased expression of members of the death receptor pathway, the TNFα superfamily and their receptors, but also of TNFα agonists may imply regulated activation of pro-apoptotic activity [26-30]. Up-regulation of TRAIL, DR5, and FAS ligand acting via FADD, the universal adaptor protein known domain-containing members of the TNF receptor superfamily, would successively activate caspases 8, 10 and 3 as well as possible G1-S cell cycle progression [27]. However, the antagonists TNFAIP3, FLIP and cIAP, which respectively inhibit apoptosis via TRAF6, caspases 8, 9, 10 and TRAF-2 directly or indirectly are also prominent amongst the up-regulated genes [29-32].

Moreover, several other key proteins for the cell cycle and apoptosis are affected. Thus CDKN1A (P21, WAF, WAF1 or CIP1) which plays a pivotal role in inhibiting cell cycle progression at several points in response to DNA damage [33], is up-regulated, as are FOXO1A and SMAD 2 (Additional file 1) and 3 (Additional file 2), which act together to increase CDKN1A activity [34,35]. Conversely, other genes that inhibit cell cycle progression are down-regulated. These include SKP2, the F-box receptor that interacts with p19 and the CDK2/cyclin A to prevent entry into G1 [36] and SFN (stratifin or 14-3-3σ) a key target of the tumour suppressor gene TP53 which acts to cause G2 arrest [37].

Five other changes of potential functional importance are of note. Firstly, a number of potentially antibacterial agents are highly induced, including LCN2 (lipocalin-2) [38,39] and PI3 (peptidase inhibitor 3, aka ELAFIN) [40], whilst MMP7 is thought to activate defensins [41]. Secondly, five key molecules involved in antigen processing and presentation (Figure 1, 2) [42] were also up-regulated and could play a role in the development of immune responses to *C. jejuni*. Thirdly, alterations in matrix metalloproteinases and leukocyte receptors would influence the inflammatory response, with MMP9 acting to facilitate neutrophil transfer by activating interleukin-8 [43] and MMP7 acting to localize them to sites of tissue damage [44]. Fourthly, the ephrin pathway (Figure 2), including Ephrin A2 and B2 receptors (EPHA2, EPHB2) and Ephrin A1 (EFNA1, Figure 3), rho kinase (ROCK2), Rac, ARP2/3, CDC42 and WASP appeared to be strongly up-regulated. This pathway is concerned with activation of cytokinetic changes that may potentially play a role in rapid restitution [45,46]. Finally, up-regulation of the folate receptor (FOLR1) may reflect preparation for reparative nucleotide synthesis dependent upon one-carbon transfer activity [47].

## Conclusion

The data we have generated using a BCE of *C. jejuni *represents a reductionist approach to determine some of the cellular responses associated with *C. jejuni *infection. However, because *C. jejuni *BCE represents a robust NF-κB inducing activity that is not only heat-stable but resistant to protease and acidic pH (pH 3) [8], these may indeed be of clinical significance if these products are shed upon *C. jejuni *infection or co-delivered through the diet. *C. jejuni *has been detected in many commercially available chicken portions [2] and clinical cases of *Campylobacter *enterocolitis are frequently associated with ingestion of partially cooked poultry meat [48].

Changes in host gene expression following *C. jejuni *BCE interestingly reflects some of the changes that are known to occur in inflammatory bowel diseases (IBD) such as ulcerative colitis, for which *C. jejuni *colitis can be considered a model, and may therefore indicate other potential targets for investigation of epithelial-derived mediators of inflammation in ulcerative colitis/IBD. Up-regulation of NF-κB is well recognized and considered a possible target of mesalazine [49,50]. Genes up-regulated by *C. jejuni *that have been associated with active ulcerative colitis/IBD include chemokines [51], such as IL8 and CCL20 (macrophage inflammatory protein 3α) [52-54] cytokines, including TNFα [55], eicosanoids [53] and elafin [56]. IL23, IL32 [57-59] and receptors such as interferon-γ receptor, and TLR2 [60] have all been demonstrated to be altered here (Table 2, Additional file 1). Activation of pro-apoptotic pathways involving the TNF superfamily and death domain signalling pathway have been reported to be up-regulated in colonic enterocytes isolated from patients with ulcerative colitis, from which C-IAP2 (BIRC3) has been proposed as a disease marker [61], whilst the leukocytes serine anti-proteinase elafin has recently been identified as a candidate biomarker for ulcerative colitis but with attenuated induction in Crohn's disease [56]. Thus, the data we report here include a number of pathways and mediators that may be realistic anti-inflammatory therapeutic targets to prevent or reduce the activity of *C. jejuni *colitis or ulcerative colitis. These targets include mechanisms for chemoattraction of inflammatory cells, cellular processes associated with repair and the processes associated with apoptosis, as well as NF-κB itself, the utilization of which can be investigated by intervention studies in model systems and humans.

## Competing interests

The authors declare that they have no competing interests.

## Authors' contributions

KM conceived the study, designed, co-ordinated, interpreted the experiments, and co-wrote the manuscript. IC determined the characteristics of the BCE, contributed to experimental design, interpretation of data, and to the writing of the manuscript. ML drafted the original manuscript, performed some of the cytokine analysis and contributed to analysis of data. PC performed the analysis of transcriptomics by Bioconducter and IPA. JS performed the BCE induction experiment. ED performed RQ-PCR analysis. FM and PC analysed components of BCE. CH Co-wrote the manuscript and interpreted the data. All authors read contributed to and approved the final manuscript.

## Supplementary Material

Additional file 1 **Complete listed of significantly regulated genes induced by *C. Jejuni* BCE.** File contains all genes identified by the Bioconductor and IPA programmes as significantly regulated (S score ≤ -2.5 or ≥ 2.5). All genes are shown together with their synonym, description, Genbank name, S score, network allocation, location, family, Entrez ID for Human, Mouse and Rat, and NCBI Entrez Gene web-link. Network 1 is displayed in  additional file 3, network 2 is displayed in  additional file 4, network 3 is displayed in figure 3, and network 4 is displayed in additional file 2. Click here for file

Additional file 2 **IPA generated cell movement associated gene network.** All 35 focus genes in this pathway are significantly up or down-regulated. Labeling of Network is similar to that of figure 3. Genes with an S score of ≥ 7 are shown in red and those with an S score between 2.5–7 are shown pink. Down-regulated genes with an S score between -2.5 and -7 are shown green. Click here for file

Additional file 3 **IPA generated MYC associated gene network.** All 35 focus genes in this pathway are significantly up or down-regulated. Labeling of Network is similar to that of figure 3. Genes with an S score of ≥ 7 are shown in red and those with an S score between 2.5–7 are shown pink. Down-regulated genes with an S score between -2.5 and -7 are shown green. Click here for file

Additional file 4 **IPA generated cell death associated gene network.** All 35 focus genes in this pathway are significantly up or down-regulated. Labeling of Network is similar to that of figure 3. Genes with an S score of ≥ 7 are shown in red and those with an S score between 2.5–7 are shown pink. Down-regulated genes with an S score between -2.5 and -7 are shown green. Click here for file
