# Supplementary figures and images for: Induction of a chemoattractant transcriptional response by a Campylobacter jejuni boiled cell extract in colonocytes
Source: BMC Microbiol. 2009 Feb 4;9:28. doi: 10.1186/1471-2180-9-28 (PMC2672935; doi:10.1186/1471-2180-9-28)

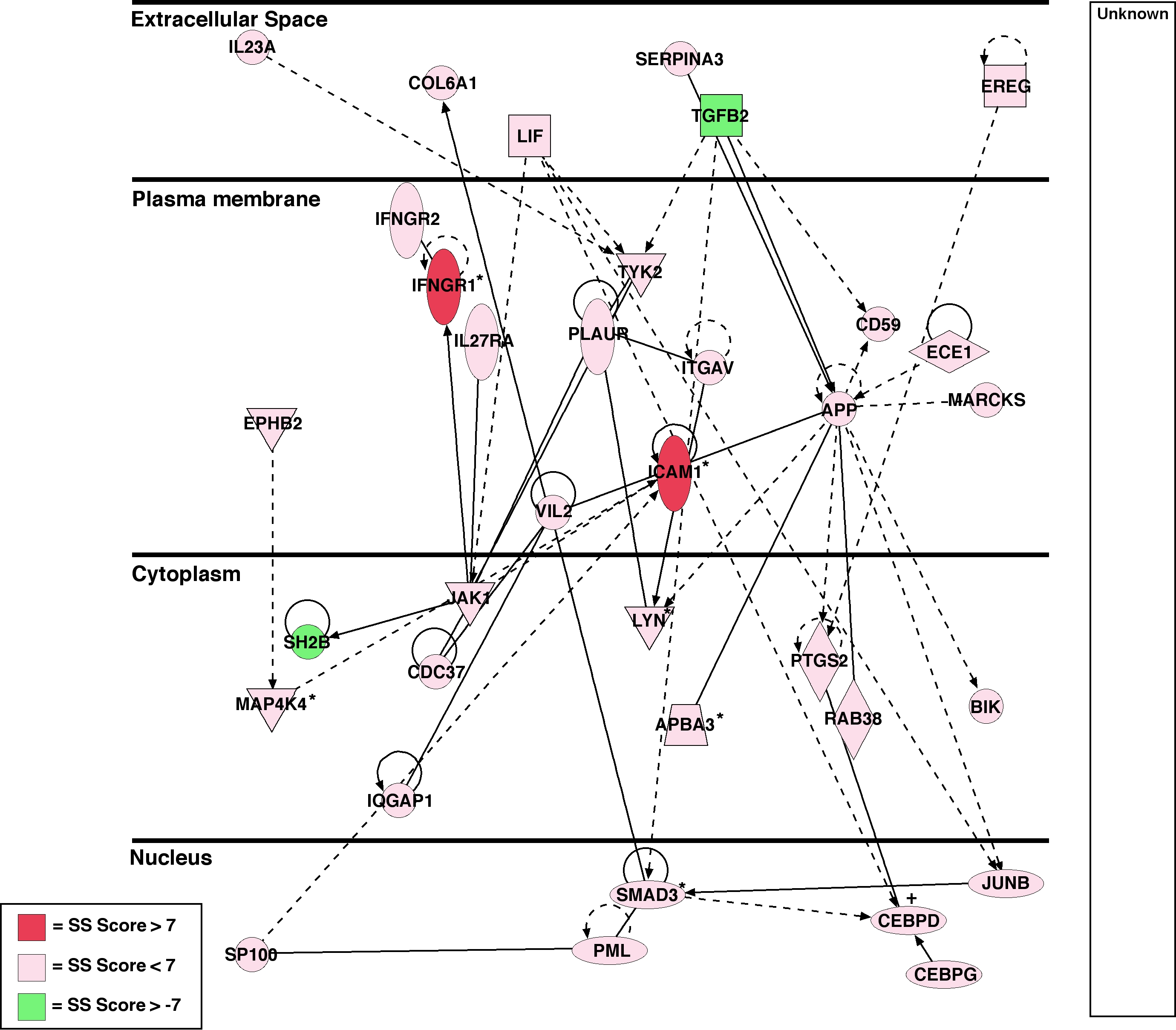

Supplement: Additional file 2 — IPA generated cell movement associated gene network. All 35 focus genes in this pathway are significantly up or down-regulated. Labeling of Network is similar to that of figure 3. Genes with an S score of ≥ 7 are shown in red and those with an S score between 2.5–7 are shown pink. Down-regulated genes with an S score between -2.5 and -7 are shown green. [file 1471-2180-9-28-S2.jpeg]

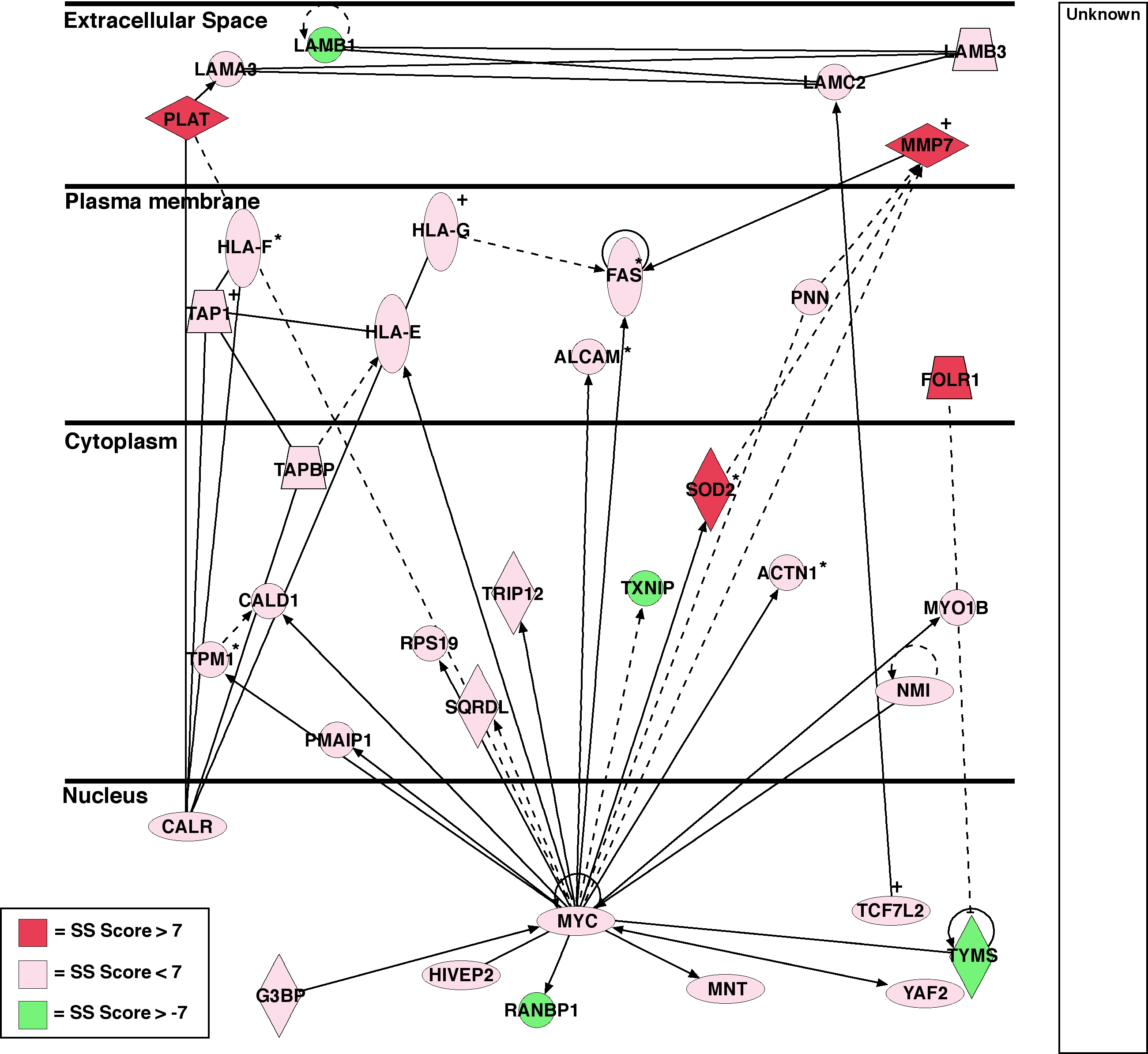

Supplement: Additional file 3 — IPA generated MYC associated gene network. All 35 focus genes in this pathway are significantly up or down-regulated. Labeling of Network is similar to that of figure 3. Genes with an S score of ≥ 7 are shown in red and those with an S score between 2.5–7 are shown pink. Down-regulated genes with an S score between -2.5 and -7 are shown green. [file 1471-2180-9-28-S3.jpeg]

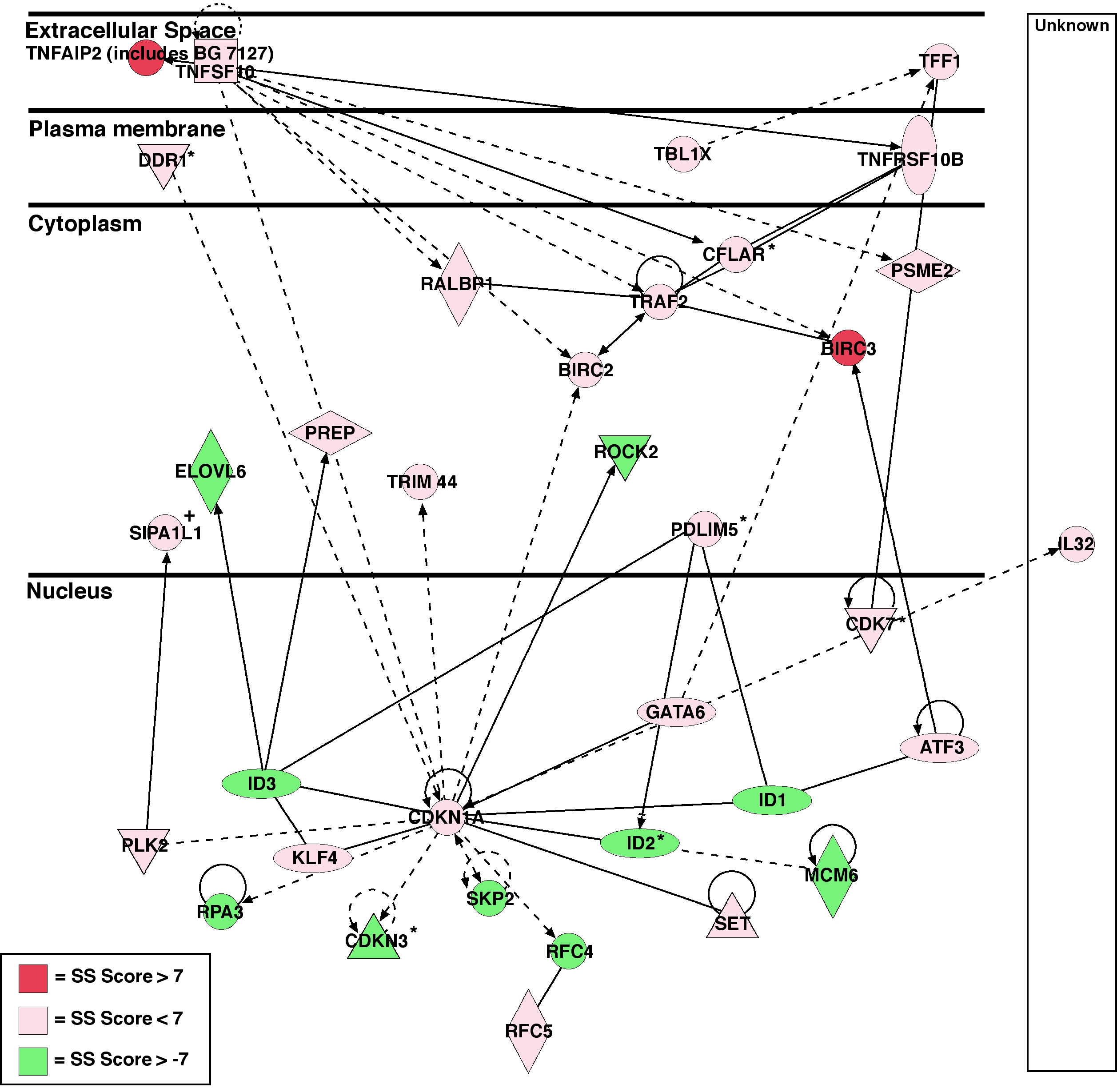

Supplement: Additional file 4 — IPA generated cell death associated gene network. All 35 focus genes in this pathway are significantly up or down-regulated. Labeling of Network is similar to that of figure 3. Genes with an S score of ≥ 7 are shown in red and those with an S score between 2.5–7 are shown pink. Down-regulated genes with an S score between -2.5 and -7 are shown green. [file 1471-2180-9-28-S4.jpeg]
